# Cerebral microhemorrhages in a mouse model of sickle cell disease

**DOI:** 10.1093/jscdis/yoag015

**Published:** 2026-03-09

**Authors:** Yu-Han Hung, Chuo Fang, Donghy Lee, Jiamin Yan, Stacy Kiven, Jihua Liu, Seung Min Kim, Annlia Paganini-Hill, David H Cribbs, Kalpna Gupta, Mark Fisher

**Affiliations:** Department of Neurology, University of California, Irvine, Orange, CA, 92868, United States; Department of Neurology, University of California, Irvine, Orange, CA, 92868, United States; Department of Neurology, University of California, Irvine, Orange, CA, 92868, United States; Department of Neurology, University of California, Irvine, Orange, CA, 92868, United States; Division of Hematology/Oncology, Department of Medicine, University of California, Irvine, Irvine, CA, 92697, United States; Institute for Memory Impairments and Neurological Disorders, University of California, Irvine, Irvine, CA, 92697, United States; Department of Neurology, University of California, Irvine, Orange, CA, 92868, United States; Department of Neurology, Veterans Health Service Medical Center, Seoul, 05368, South Korea; Beckman Laser Institute, University of California, Irvine, Irvine, CA, 92697, United States; Institute for Memory Impairments and Neurological Disorders, University of California, Irvine, Irvine, CA, 92697, United States; Division of Hematology/Oncology, Department of Medicine, University of California, Irvine, Irvine, CA, 92697, United States; Department of Neurology, University of California, Irvine, Orange, CA, 92868, United States; Beckman Laser Institute, University of California, Irvine, Irvine, CA, 92697, United States; Department of Pathology and Laboratory Medicine, University of California, Irvine, Orange, CA, 92868, United States

**Keywords:** sickle cell disease, cerebral microhemorrhages, mast cells, small vessel disease

## Abstract

**Objectives:**

Stroke in sickle cell disease (SCD) is often attributed to large vessel involvement in the disorder, whereas the contribution of cerebral microvascular disease has been less explored. In this study, we investigated the formation of cerebral microvascular lesions and the involvement of mast cells in a humanized SCD mouse model.

**Methods:**

We studied hemorrhagic microvascular disease in a well-characterized mouse model of humanized transgenic sickle (HbSS-BERK) expressing >99% human sickle hemoglobin (HbS) and a control (HbAA-BERK) mouse model expressing normal human hemoglobin A (HbA). Mouse brains were analyzed by Prussian blue staining to detect cerebral microhemorrhage (CMH) formation. Mast cell identification was performed by toluidine blue staining.

**Results:**

SCD brain sections exhibited approximately 86% more CMH than controls (mean ± SE of 1.17 ± 0.22 vs. 0.63 ± 0.13 number/cm^2^, *P* = .02). Mast cells were positively correlated with CMH number in SCD mice (Spearman *r* = 0.42, *P* < .05), but not in control mice.

**Conclusion:**

SCD mice demonstrated significantly increased CMH load compared with control mice, and SCD microhemorrhages were associated with the number of mast cells. These findings highlight the significance of cerebral microvascular disease in SCD and imply that cerebral mast cells may be a novel therapeutic target in SCD.

## GRAPHICAL ABSTRACT

**Figure:**
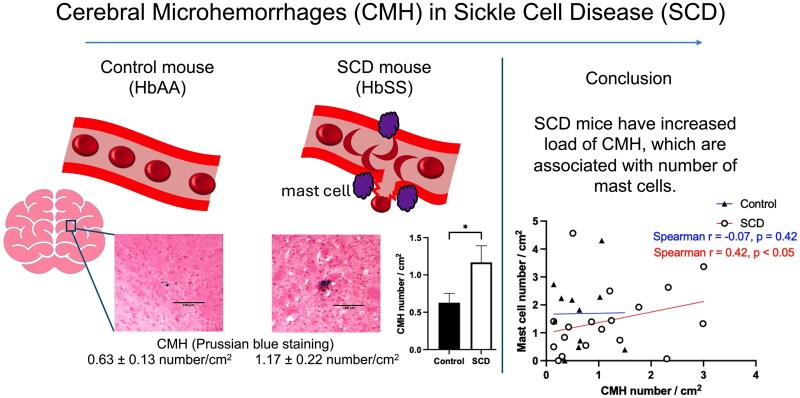

## INTRODUCTION

Sickle cell disease (SCD) is a heritable hemoglobinopathy affecting one in every 365 African Americans and 20 million people worldwide.^1^ It is caused by a point mutation on the β-globin gene found on chromosome 11, resulting in deoxygenated sickle cell hemoglobin (HbS). Clinically apparent stroke occurs in around 11% of individuals with SCD before age 20, with the risk rising to 24% by the age of 45.^2^

In individuals with SCD, ischemic stroke predominates in childhood,^3^^,^^4^ while hemorrhagic stroke becomes more common in adolescence and adulthood, contributing to the disease burden in older individuals.^5^ This indicates the importance of characterizing non-ischemic cerebrovascular complications, such as cerebral microhemorrhages (CMH), particularly in adult models of SCD.^6^

CMH are the pathological substrate for cerebral microbleeds (CMB), which appear as focal hemosiderin/iron deposits on MRI and are associated with cognitive impairment, as well as ischemic and hemorrhagic stroke.^7^^,^^8^ CMB indicate impaired small vessel integrity and are associated with small vessel disease (SVD) including cerebral amyloid angiopathy. Notably, persons with SCD may exhibit evidence of cerebral SVD even prior to the onset of symptoms.^9^

Mast cells are a type of tissue resident granulocyte derived from myeloid stem cells and are a component of the immune and neuroimmune systems.^6–8^ Hyperactive mast cells have been associated with various neuroinflammatory conditions of the central nervous system including stroke,^10–12^ blood-brain barrier (BBB) injury, vasogenic edema, hemorrhage formation, and can recruit other immune cells amplifying inflammatory response.^13^^,^^14^ Mast cell activation is a part of the neurogenic inflammatory pathway, and their activation includes degranulation, synthesis of lipid mediators, and cytokine release.^10^^,^^15^^,^^16^ In SCD, mast cells usually act as pro-inflammatory effector cells that produce and release granules during pain activation.^15–18^

It is widely recognized that patients with SCD have higher risk of developing neurological complications. In this study, we examined the development of cerebral microvascular lesions as well as the role of mast cells in a mouse model of SCD. We hypothesized that SCD in mice leads to increased load of CMH, which are positively associated with mast cells.

## MATERIALS AND METHODS

### Mice

We used a well-characterized mouse model of humanized transgenic SCD (HbSS-BERK) and control (HbAA-BERK) mice.^17^^,^^19^ HbSS-BERK expresses human α and βS hemoglobins (>99% HbS), while HbAA-BERK produces normal human α and βA hemoglobins (HbA); both are deleted for mouse α and β globins on a mixed genetic background. Seven to nine-month-old male mice (*N* = 18 HbSS, N = 11 HbAA) were examined in this study. All experimental procedures followed the NIH Guide for the Care of Use of Laboratory Animals and were approved by the Institutional Animal Care and Use Committee at the University of California, Irvine (UCI).

### Tissue harvest and brain sectioning

Mice were euthanized under inhaled isoflurane, followed by cardiac perfusion with ice-cold phosphate-buffered saline (PBS). Brains were collected, fixed in 10% formalin overnight, and then passed through 15% and 30% sucrose/PBS solutions prior to storage at −70 °C. We used a freezing microtome to obtain 20-µm coronal sections.

### Quantification of CMH and mast cells

A total of 32 coronal brain sections 20 microns thick were taken from the olfactory bulb to the cerebellum of each mouse (at 10-section intervals) for analysis. Prussian blue staining for hemosiderin was performed by the Department of Pathology & Laboratory Medicine at UCI Medical Center. Sections adjacent to those collected for CMH detection were used for toluidine blue staining to detect mast cells. In brief, sections were stained with 5% potassium hexacyanoferrate trihydrate (Sigma-Aldrich, St. Louis, MO, United States) and 10% hydrochloric acid (Sigma-Aldrich, St. Louis, MO, United States) for 30 min. After rinsing with water, they were counterstained with nuclear fast red, dehydrated, and coverslipped. Sections for mast cell staining were treated with freshly prepared 0.5% toluidine blue (Catalog #: 01804, Chem-Impex International Inc., Wood Dale, IL, United States), distilled water, 100% alcohol, and hydrochloric acid for 8 minutes, and were then rinsed with water, dehydrated, and coverslipped. CMH was quantified by detection and imaging at 20× magnification, and mast cells were detected by imaging at 20× and 60× magnification via a light microscope by a blinded observer. The location of CMH and mast cells in each brain region were recorded for every mouse. Whole slide images were scanned to quantify the total area of the brain section using the National Institute of Health (NIH) ImageJ software 1.53k. The number of CMH and mast cells were then adjusted to the total area of the brain sections per animal.

### Statistical analysis

The data were analyzed with GraphPad Prism 9 software (GraphPad Software, La Jolla, CA, United States). Results are presented as mean ± SEM. Comparison of means was performed with Welch’s *t*-test for independent groups and a one-sided *P*-value testing the null hypothesis of equal or smaller means of CMH and mast cell number vs the alternative hypothesis of a larger mean in the SCD mice. Spearman’s rank correlation coefficients (r) were calculated to determine the association between CMH load and mast cell number and tested for a positive correlation. A one-sided *P* value of less than .05 was considered statistically significant.

## RESULTS

### SCD promotes CMH formation in the HbSS mouse model

**Figure 1.**
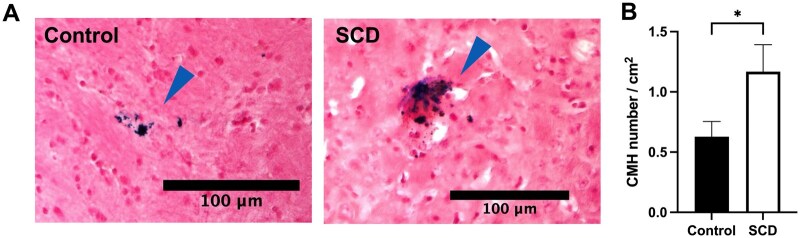
Sickle cell disease (SCD) mice show increased cerebral microhemorrhage (CMH) burden. (A) Representative images of Prussian blue-positive lesions (blue arrowhead) in the brain sections from control and SCD mice. Scale bar = 100 μm. (B) SCD mice had a significantly higher CMH number per cm^2^ compared with control mice. Data shown are mean ± SEM, *N* = 11 in control (HbAA) mice, *N* = 18 in SCD (HbSS) mice, age 8.2 ± 0.1 months old. *P < .05.

Prussian blue staining was used to examine the CMH burden of the SCD (HbSS) and control (HbAA) mice. Representative images of Prussian blue-positive lesions in the brain sections are shown in Figure 1A. CMH burden was measured by the number of Prussian blue-positive CMH adjusted to the total area of the brain sections (CMH number per cm^2^). Consistent with our hypothesis, CMH was significantly increased in SCD mice compared with control mice (mean ± SE of 1.17 ± 0.22 vs. 0.63 ± 0.13 number/cm^2^, *P* = .02) (Figure 1B). HbSS mice showed approximately 86% more CMH than control mice. CMH were most prominent in the cortical area in both SCD and control mice (Figure S1A). In addition, CMH area was larger in SCD mice, while CMH size did not significantly differ between the two groups (Figure S2).

### Mast cell number was positively correlated with CMH number in SCD mice

**Figure 2.**
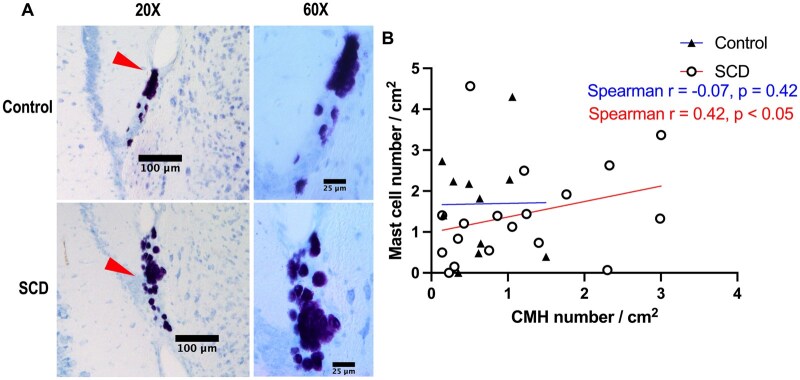
Correlations between mast cells and cerebral microhemorrhages (CMH) in sickle cell disease (SCD) mouse model. (A) Representative images of toluidine blue staining of mast cells (red arrowhead) in the brain sections from control and SCD mice. Scale bar = 100 μm in 20× magnification, and scale bar = 25 μm in 60× magnification. (B) Mast cell number was positively correlated (Spearman correlation) with CMH number in SCD mice but not in control mice. *N* = 11 in control (HbAA) mice, *N* = 18 in SCD (HbSS) mice, age 8.2 ± 0.1 months old.

To investigate mast cells, we performed toluidine blue staining in HbSS and HbAA mice. Figure 2A shows representative images of Toluidine blue-positive staining in the brain sections indicating cerebral mast cells. Mean number of mast cell per brain area did not differ significantly between control and SCD mice (mean ± SE of 1.69 ± 0.38 vs. 1.43 ± 0.28 cells/cm^2^, *P* = .30, respectively). Mast cells were most common in the brainstem/cerebellum area in both control and SCD mice (Figure S1B). We then studied the relationship between CMH and cerebral mast cells. Consistent with our hypothesis, mast cell number per cm^2^ positively correlated with CMH number per cm^2^ of brain area in SCD mice (Spearman *r* = 0.42, *P* < .05), while no significant correlation was observed in control mice (Spearman *r* = −0.07, *P* = .42) (Figure 2B).

## DISCUSSION

We demonstrate enhanced CMH formation in a mouse model of SCD (HbSS mice). In addition, number of CMH in SCD mice was positively associated with mast cell count. While previous studies have shown increased brain iron accumulation in SCD patients,^20^ this is the first study that highlights CMH development in a mouse model of SCD.

Mast cell activation is thought to play a significant role in ischemia-reperfusion injury.^21^ Previous studies suggest that mast cell granules containing tryptase, which along with extracellular traps impair the BBB by interacting with endothelium and nerve fibers in SCD mice.^15–18^ Mast cells, activated by the chronic inflammatory environment, release mediators such as histamine and cytokines, which can exacerbate inflammation and vascular permeability,^22^^,^^23^ weaken the BBB, and increase the risk of hemorrhage. Although mast cell number was not elevated in SCD mice compared with control mice in this study, their activation may play an important role in CMH pathogenesis. The release of vasoactive mediators and formation of mast cell extracellular traps can directly impair vascular permeability and endothelial stability,^15^^,^^24^ and are an appropriate target for future investigations. Mast cells also promote fibrinolysis^25^ and activate matrix metalloproteinases, which may further impair the BBB.^26–28^ Elevated plasma histamine levels in SCD patients during vaso-occlusive crises are consistent with the role of mast cells in SCD pathophysiology.^29^ Together, these observations suggest that brain-resident mast cells may influence microvasculature and contribute to CMH formation.

Although mast cells are classically viewed as tissue-resident immune cells, it is important to clarify that they do not circulate as mature cells. Instead, they derive from hematopoietic stem cells and enter tissues as precursors, where they undergo final differentiation and maturation locally.^16^ This process is highly influenced by the regional microenvironment, leading to substantial heterogeneity in mast cell phenotype and function across different organs and disease contexts. In our study, mast cells observed in brain tissues are likely of resident origin, reflecting a locally adapted phenotype potentially distinct from peripheral counterparts. This distinction is particularly relevant in the context of therapeutic targeting, as interventions aimed at modulating mast cell activity must account for the tissue-specific behavior and signaling of mast cells in the CNS during SCD. Although normally restricted to the meninges, mast cells can infiltrate brain regions under pathological conditions.^13^

Our study has some limitations. First, only male mice were studied. Female mice were excluded due to the fluctuating hormone levels (eg, estrogen, progesterone) throughout the estrous cycle, which can impact vascular integrity and inflammatory response;^30^ male mice were used to minimize this variability. Future research including female animals is essential to determine the influence of sex on cerebrovascular injury and immune cell dynamics. Age is a key determinant of cerebrovascular health, and aging is known to exacerbate vascular dysfunction and inflammatory responses. The present study utilized 7-9-month-old mice, which are considered middle-aged.^31^ Age-related changes in vascular integrity and mast cell activity might have contributed to the observed phenotype. Future studies including younger and older cohorts will help clarify the role of aging in modulating CMH and mast cell dynamics in SCD. Moreover, we cannot rule out that mast cell presence and activation are a consequence, rather than a cause, of CMH development. In addition, we cannot rule out endothelial erythrophagocytosis of sickle cells contributing to CMH formation.^32^ While Prussian blue staining effectively detects subacute and chronic CMH, it can be influenced by sectioning variability and potential artifacts inherent to iron-sensitive histological techniques.^33^

## CONCLUSION

Our findings indicate increased CMH formation in HbSS mice, and a significant association of CMH with mast cell number in these mice. Targeting mast cells may be a potentially useful strategy to address cerebral small vessel disease in SCD.

## Supplementary Material

yoag015_Supplementary_Data

## Data Availability

Data are available upon reasonable request.
