# Supplementary material for: Cerebral microhemorrhages in a mouse model of sickle cell disease
Source: J Sick Cell Dis. 2026 Mar 9;3(1):yoag015. doi: 10.1093/jscdis/yoag015 (PMC13020912; doi:10.1093/jscdis/yoag015)
Supplement: yoag015_Supplementary_Data [file yoag015_supplementary_data.zip › CMH in SCD Supplementary Data.docx]

**Cerebral Microhemorrhages in a Mouse Model of Sickle Cell Disease**

Yu-Han Hung, PhD^1^, Chuo Fang, PhD^1^, Donghy Lee, BS^1^, Jiamin Yan, BS^1^, Stacy Kiven, MS^2^, Jihua Liu, MD^3^, Seung Min Kim, MD^1, 4^, Annlia Paganini-Hill, PhD^5^, David H. Cribbs, PhD^3^, Kalpna Gupta, PhD^2^, and Mark Fisher, MD^1, 5, 6^ *

^1^Department of Neurology, University of California, Irvine, CA, USA.

^2^Division of Hematology/Oncology, Department of Medicine, University of California, Irvine, CA, USA.

^3^Institute for Memory Impairments and Neurological Disorders, University of California, Irvine, CA, USA.

^4^Department of Neurology, Veterans Health Service Medical Center, Seoul, South Korea.

^5^Beckman Laser Institute, University of California, Irvine, CA, USA. ^6^Department of Pathology and Laboratory Medicine, University of California, Irvine, CA, USA.

***Correspondence to:**

Mark Fisher, MD
Department of Neurology

UC Irvine Medical Center

101 The City Drive South, Shanbrom Hall, Room 121

Orange, CA, 92868, USA

Email: [mfisher@hs.uci.edu](mailto:mfisher@hs.uci.edu)

Phone: (714) 456-6856

Fax: (714) 456-6573

**Supplementary Data**

**
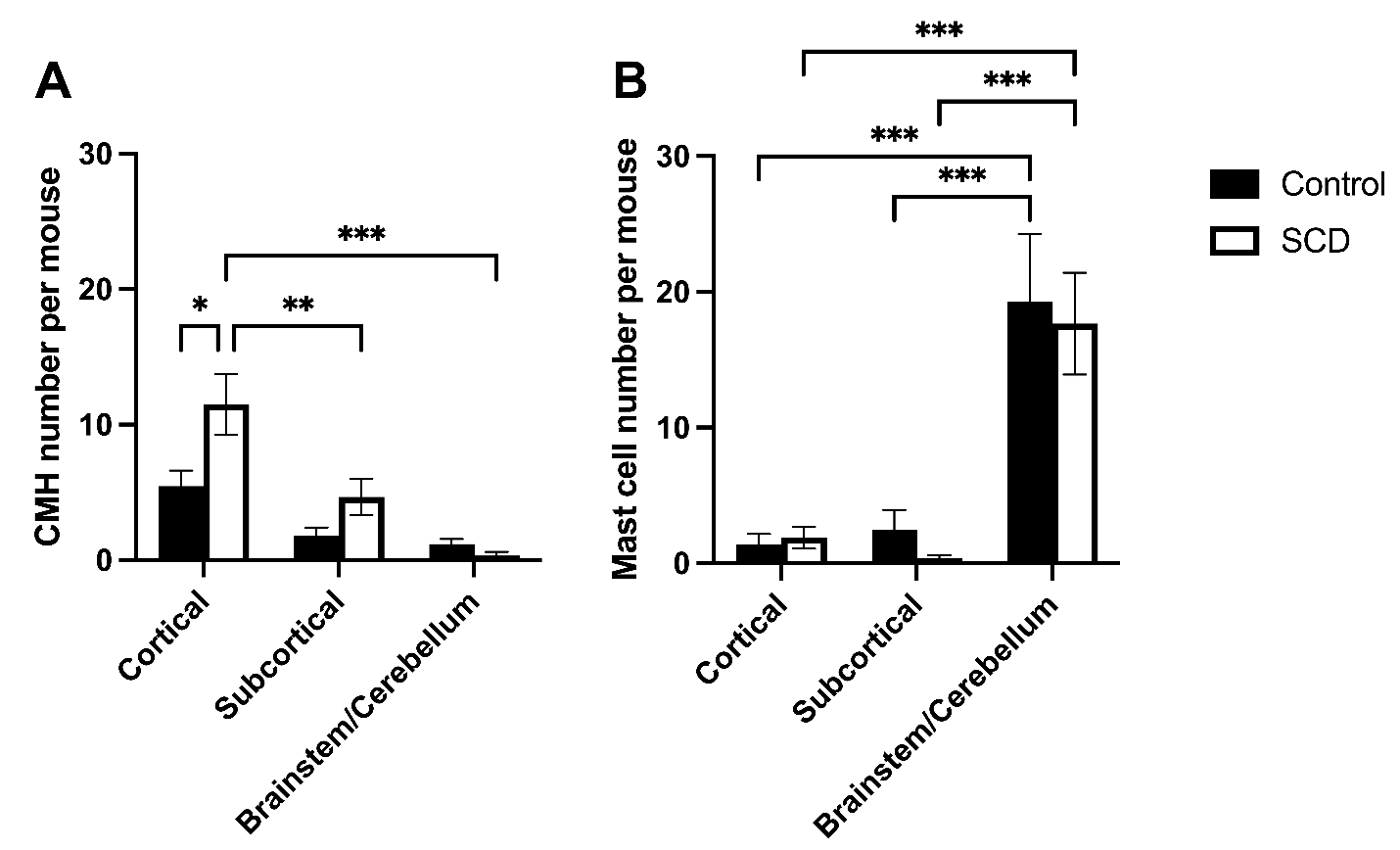
**

Figure S1. (A) CMH number and (B) mast cell number in cortical, subcortical, and brainstem/cerebellum areas for control and SCD mice. CMH are most abundant in the cortical area, whereas mast cells tended to be more common in the brainstem/cerebellum area. Data are presented as mean ± SEM. Statistical comparisons were performed using two-way ANOVA followed by two-tailed Holm-Šídák-adjusted pairwise analysis. N=11 Control (HbAA) mice, and N=18 SCD (HbSS) mice. **p<0.05*, ***p<0.01*, ****p<0.001*.


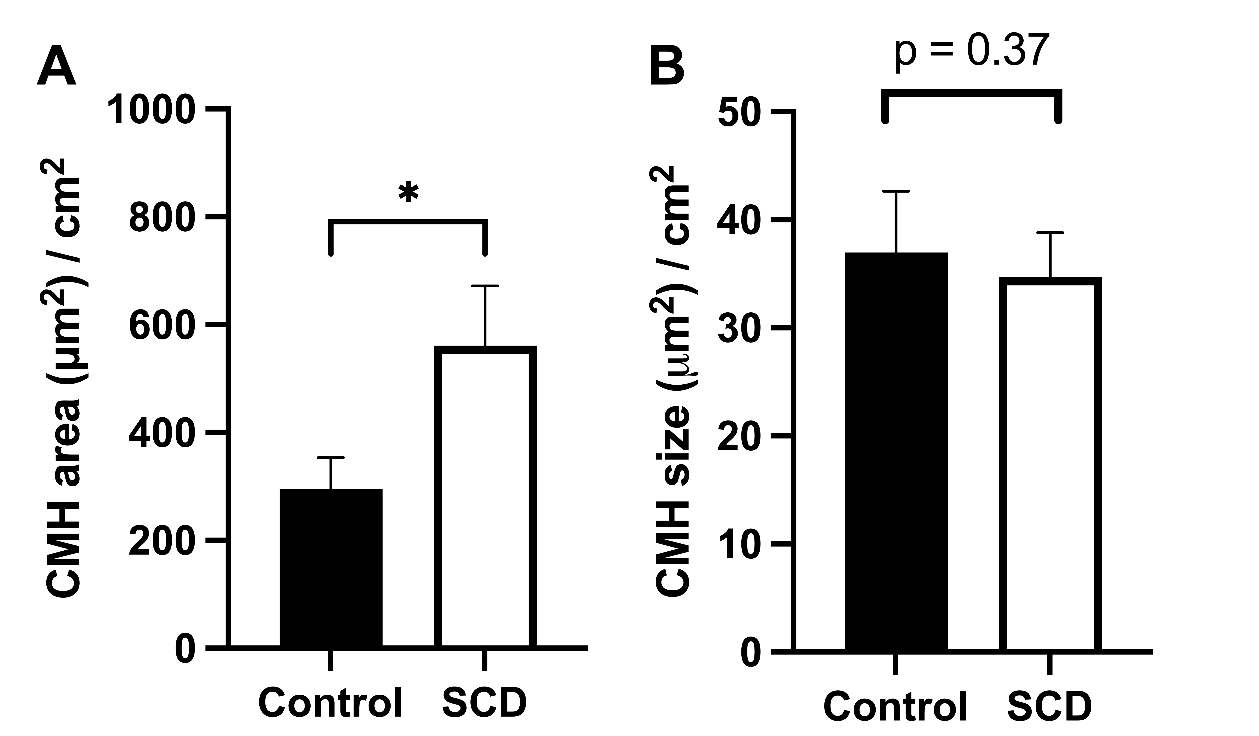


Figure S2. Quantification of (left) CMH area and (right) CMH size in control and SCD mice. CMH area, but not CMH size, differed between control and SCD mice. Data shown are mean ± SEM, with 32 sections per mouse. **p<0.05*.
