# Supplementary figures and images for: Cerebral microhemorrhages in a mouse model of sickle cell disease
Source: J Sick Cell Dis. 2026 Mar 9;3(1):yoag015. doi: 10.1093/jscdis/yoag015 (PMC13020912; doi:10.1093/jscdis/yoag015)

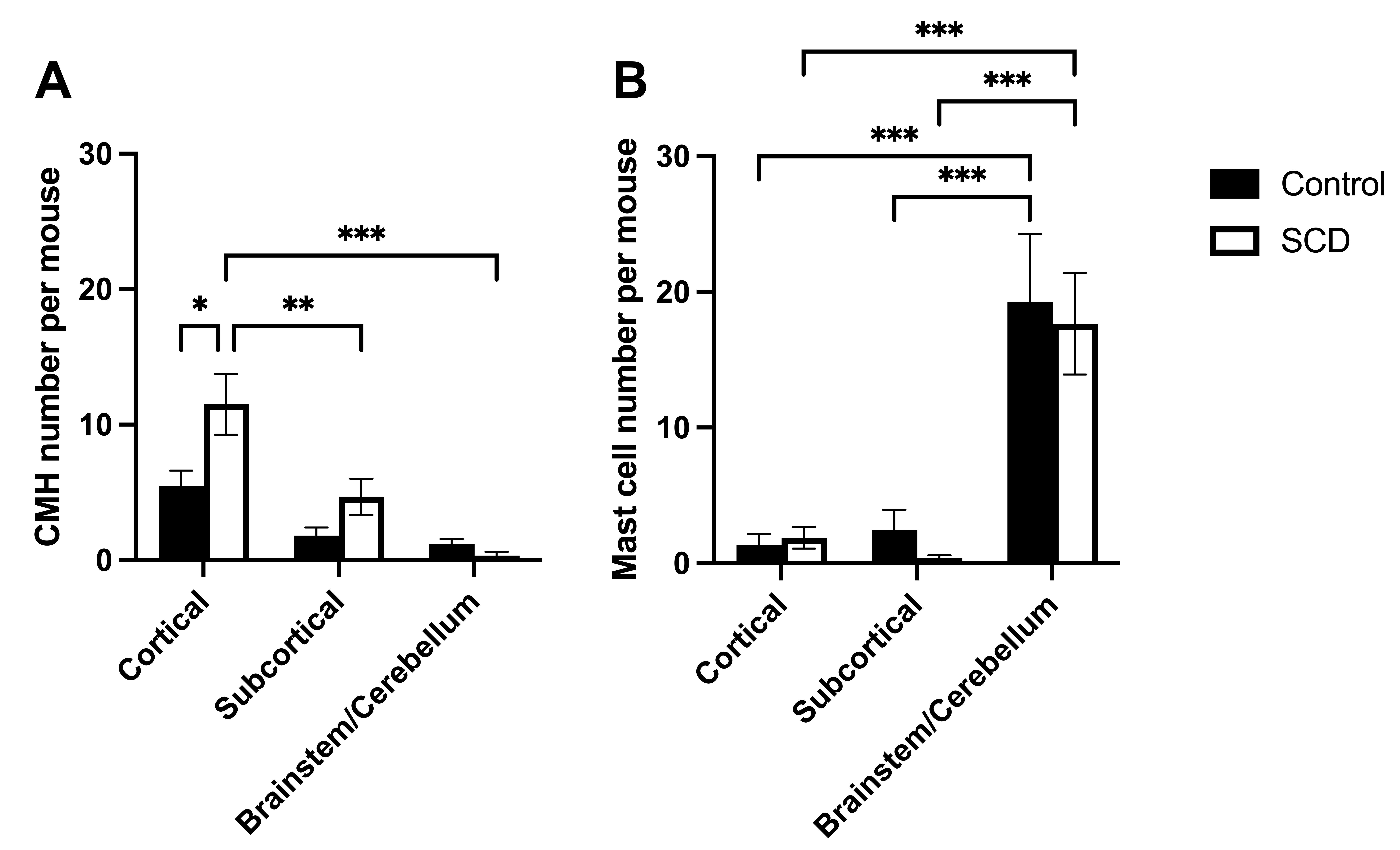

Supplement: yoag015_Supplementary_Data [file yoag015_supplementary_data.zip › CMH in SCD Figure S1.tiff]

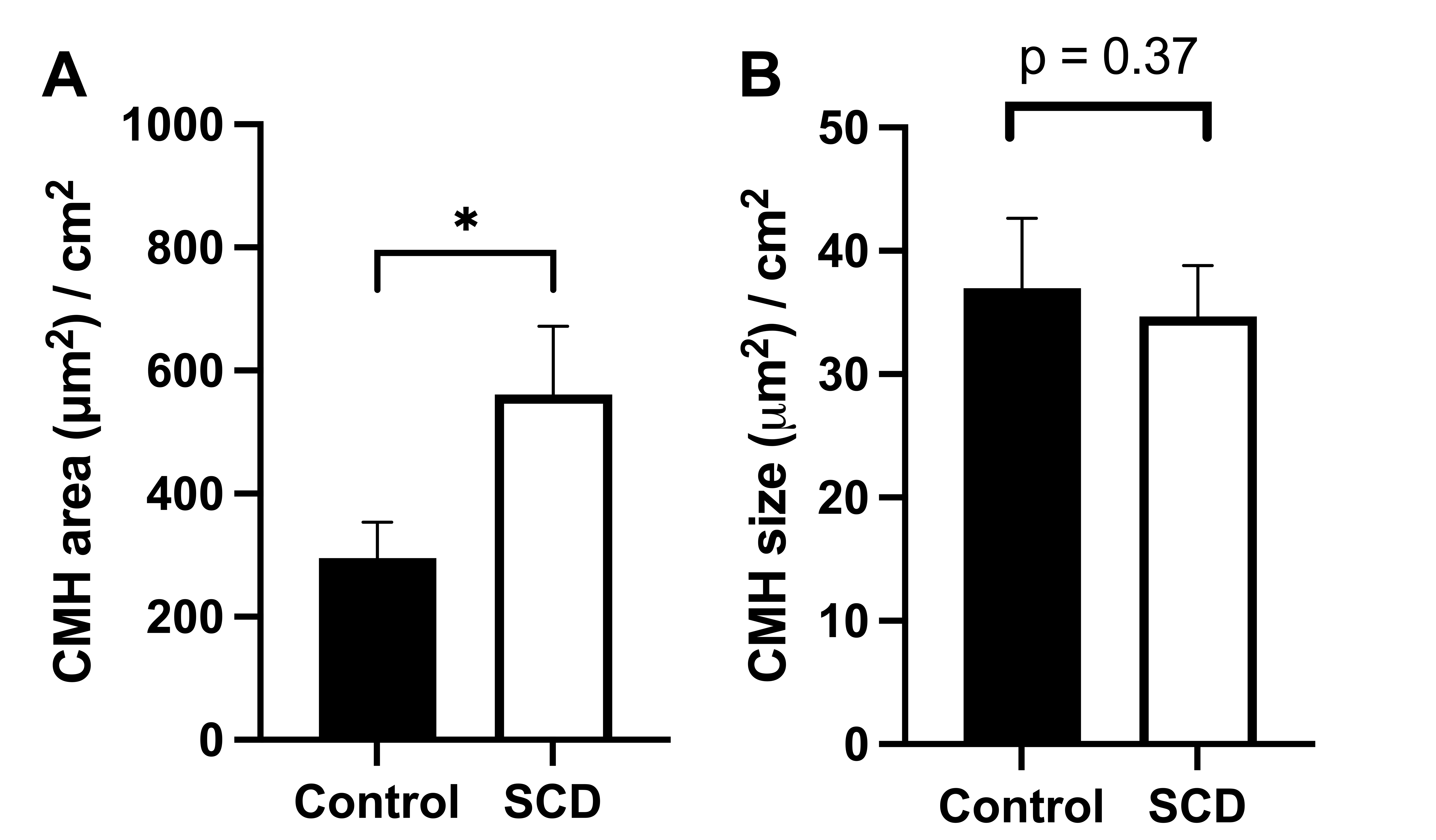

Supplement: yoag015_Supplementary_Data [file yoag015_supplementary_data.zip › CMH in SCD Figure S2.tiff]
